# Fatal Gets More Fatal: A COVID-19 Infection With Macrophage Activation Syndrome

**DOI:** 10.7759/cureus.25591

**Published:** 2022-06-02

**Authors:** Yucel Aydin, Bhavya Vemuri, Jackeline P Vajta Gomez, Pavan K Challa, He Zhang

**Affiliations:** 1 Internal Medicine, Saint Mary's Hospital, Waterbury, USA; 2 Pulmonary and Critical Care Medicine, Saint Mary's Hospital, Waterbury, USA

**Keywords:** macrophage activation syndrome (mas), acute respiratory distress syndrome [ards], cytokine storm, covid-19, sars-cov-2

## Abstract

Coronavirus disease 2019 (COVID-19) continues to be fatal despite advances in the understanding of characteristics of Severe Acute Respiratory Syndrome Coronavirus 2 (SARS-CoV-2), global prevention strategies, new anti-viral treatments, and worldwide vaccination programs. The exact underlying mechanism through which SARS-CoV-2 leads to acute respiratory distress syndrome (ARDS) resulting in intensive care unit admission, mechanical ventilation, and eventually death remains elusive. Cytokine storm is one of the most favorable mechanisms that scientists show remarkable interest to target in randomized clinical trials with promising outcomes. Macrophage activation syndrome (MAS), the most serious form of cytokine storm, requires early recognition and treatment regardless of etiology. Here, we report a 59-year-old gentleman with a COVID-19 infection complicated by MAS. Our aim is to increase awareness of this condition among health care providers as it necessitates prompt diagnosis and treatment due to an extremely poor prognosis.

## Introduction

To date, over 500 million people were reported to have COVID-19 caused by SARS-CoV-2 all over the world since December 12, 2019, when a group of patients began to experience shortness of breath and fever in Wuhan, China [1,2]. Even though our grasp of this disastrous disease has been expanding, morbidity and mortality rate stayed significantly high until the development of prevention and treatment strategies, more important, the development of effective vaccines against it. While male gender, race, advanced age, obesity, and other comorbidities including immunosuppression are considered to be at high risk for severe COVID-19 infection, the exact mechanism by which COVID-19 causes such a rate of mortality and morbidity is unclear [3].

Cytokine storm and the exaggerated immune response is one of the major proposed mechanisms resulting in extensive bilateral lung damage called severe acute respiratory syndrome (SARS) in the setting of SARS-CoV-2 infection. MAS is the severest form of cytokine storm which is identified by overstimulation of cytotoxic lymphocytes and macrophages that is generally precipitated by genetic factors, neoplasms, and a variety of infections [4]. Here, we present a 59-year-old male with COVID-19 complicated by MAS leading to death. Our aim is to increase recognition of MAS, especially in the setting of COVID-19 among physicians as to why its treatment differs.

## Case presentation

**Figure 1:**
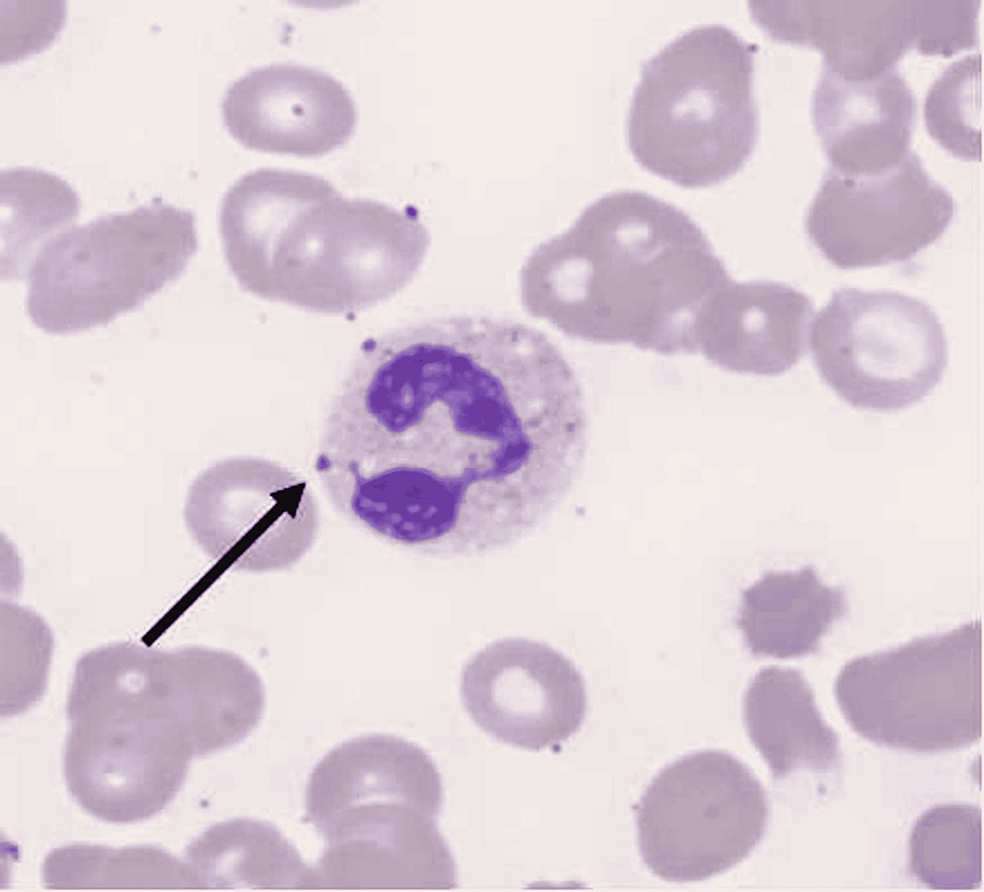
Peripheral smear on the day of admission. Arrow indicates Döhle bodies in neutrophils.

A 59-year-old African American male with morbid obesity presented with confusion, shortness of breath, dry cough, and loss of taste for nine days. On arrival, he was found to have a heart rate of 140/min, respiratory rate of 26/min, a temperature of 38.8 °C, oxygen saturation of 87% on room air with subsequent improvement to 92% on 2L nasal cannula, and blood pressure of 124/83 mmHg. On physical exam, he was significantly lethargic, confused, oriented only to the person, and in severe respiratory distress with bilateral crackles on the lung auscultation. SARS-CoV-2 was positive per PCR testing and plain chest radiography revealed bilateral patchy infiltrates, consistent with COVID-19 pneumonia. Other laboratory findings were as shown in Table 1. On day 1 of admission, a peripheral blood smear revealed visible toxic granulation and blue inclusions (Döhle bodies) inside the neutrophils (Figure 1).

**Table 1:** Laboratory values of the patient on admission and day 7.

Reference	On admission	Day 7
WBC (4.0-10.5 k/µL)	2.1	0.6
Hemoglobin (13.5-18.0 g/dL)	10.4	7.8
Platelets (150-450 k/µL)	121	32
Creatinine (0.7-1.3 mg/dL)	6.0	6.8
AST (13-39 U/L)	221	664
ALT (7-52 U/L)	59	138
CRP (<0.3 mg/dL)	20.1	10.3
D-Dimer (<231 D-DU ng/mL)	1,282	12,689
Fibrinogen (204-431 mg/dL)	>700	241
Ferritin (23.9-336.2 ng/mL)	5,860	>7,500
Interleukin-2 (175.3-858.2 pg/mL)	Not done	4,473.8

**Figure 2:**
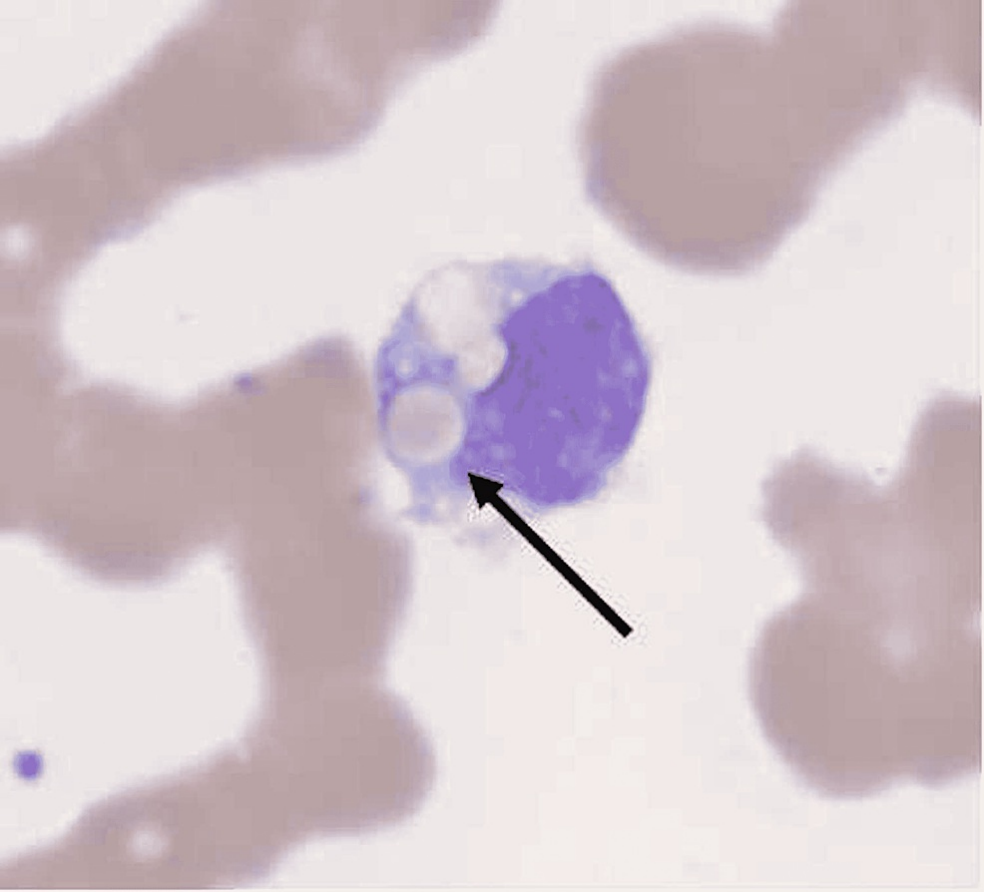
Peripheral smear on day 7. Arrow indicates hemophagocytosis by a macrophage.

The patient was subsequently intubated due to a rapid decline in oxygen saturation with labored breathing and tachypnea of 50 breath/minute (bpm) as he was non-adherent on high flow nasal cannula (HFNC) due to altered mentation. The patient developed septic shock with multiorgan failure requiring multiple vasopressors for hemodynamic support. The clinical course was complicated with atrial flutter requiring cardioversion plus amiodarone drip infusion and oliguric acute kidney injury warranting hemodialysis. In addition, he developed superimposed infections including Pseudomonas aeruginosa, Bordetella parapertussis, and disseminated Candidiasis secondary to Candida albicans. HIV-1 antibody tested positive. Repeat peripheral blood smear due to worsening pancytopenia demonstrated hemophagocytosis on day 7 (Figure 2). Unfortunately, the patient deceased due to persistent multiorgan failure and hemodynamic deterioration on the same day.

## Discussion

Severe COVID-19 infection is characterized by the presence of pneumonia plus hypoxia in patients with positive SARS-CoV-2 PCR. A group of individuals with severe COVID-19 infection subsequently requires intensive care unit (ICU) level of care and mechanical ventilation due to worsening hypoxia despite maximum non-invasive oxygen support including HFNC. Since the severity of COVID-19 infection has been shown to be directly proportional to the severity of the cytokine storm, trending inflammatory markers including complement reactive protein (CRP), fibrinogen, lymphocyte count, and ferritin give an idea to physicians about disease progression or the response to ongoing treatment. Initial peripheral smear on the day of admission revealed Döhle bodies that are small, round or oval, basophilic structures locating the periphery of the neutrophils. Döhle bodies are seen in bacterial infections, burns, trauma, and reportedly in COVID-19 [5].

Diagnosis of MAS is generally established by the presence of five out of nine following criteria; (1) fever ≥ 38.5°C, (2) bicytopenia, (3) hemophagocytosis, (4) ferritin > 500 mcg/L, (5) low/absent NK cell activity, (6) soluble CD25 elevation, (7) splenomegaly, (8) hypertriglyceridemia or hypofibrinogenemia, and (9) elevated C-X-C motif chemokine ligand 9 (CXCL9) [6]. Another diagnostic tool that was recently validated for MAS is the H score [7]. Reportedly, reduced fibrinogen and platelet count along with increased D-dimer is expected in the usual course of MAS secondary to diffuse intravascular coagulation, only elevated D-dimer is accompanied by MAS in the setting of COVID-19 infection [8]. Other cardinal findings of MAS, such as hepatosplenomegaly, are also absent in the COVID-19 [9]. Our patient had 228 points with a 96%-98% probability of MAS and met five diagnostic criteria of MAS. Unfortunately, he developed rapid multiorgan failure and expired prior to initiating MAS-specific treatment, including high-dose corticosteroid, IL-1, and six inhibitors [7].

MAS is a rare but fatal complication of inflammatory or infectious conditions including COVID-19. Multisystem inflammatory syndrome in children is excepted as a spectrum of MAS occurring mainly in children 4-6 weeks after SARS-CoV-2 exposure [10]. While MAS-like findings mostly appear after a long period of asymptomatic COVID-19 infections in children, MAS generally is an acute complication of severe COVID-19 infection in adults suggesting that the mechanism through which COVID-19 infection leads to MAS differs in children and adult populations. Although our recognition of COVID-19 has been expanding, more mechanistic studies are required to understand MAS and its treatment in patients with COVID-19 infection.

## Conclusions

COVID-19 continues to be a highly fatal disease that threatens public health with more than 6 million death throughout the world since December 2019. Cytokine storm seems to play a major role in severe COVID-19, mainly in patients developing acute respiratory distress syndrome (ARDS) that often requires ICU admission and mechanical ventilation. MAS is a rare but fatal complication of COVID-19 infection as to why the awareness of physicians for early detection and management is crucial.

## References

[1] (2022). COVID-19 Coronavirus pandemic. https://www.worldometers.info/coronavirus/.

[2] (2022). CDC museum COVID-19 timeline. https://www.cdc.gov/museum/timeline/covid19.html.

[3] Gao YD, Ding M, Dong X (2021). Risk factors for severe and critically ill COVID-19 patients: a review. Allergy.

[4] Costela-Ruiz VJ, Illescas-Montes R, Puerta-Puerta JM, Ruiz C, Melguizo-Rodríguez L (2020). SARS-CoV-2 infection: the role of cytokines in COVID-19 disease. Cytokine Growth Factor Rev.

[5] Jain S, Meena R, Kumar V, Kaur R, Tiwari U (2022). Comparison of hematologic abnormalities between hospitalized coronavirus disease 2019 positive and negative patients with correlation to disease severity and outcome [PREPRINT]. J Med Virol.

[6] Minoia F, Bovis F, Davì S (2017). Development and initial validation of the macrophage activation syndrome/primary hemophagocytic lymphohistiocytosis score, a diagnostic tool that differentiates primary hemophagocytic lymphohistiocytosis from macrophage activation syndrome. J Pediatr.

[7] Otsuka R, Seino KI (2020). Macrophage activation syndrome and COVID-19. Inflamm Regen.

[8] Schulert GS, Grom AA (2014). Macrophage activation syndrome and cytokine-directed therapies. Best Pract Res Clin Rheumatol.

[9] McGonagle D, O'Donnell JS, Sharif K, Emery P, Bridgewood C (2020). Immune mechanisms of pulmonary intravascular coagulopathy in COVID-19 pneumonia. Lancet Rheumatol.

[10] Mazer MB, Bulut Y, Brodsky NN (2022). Multisystem inflammatory syndrome in children: host immunologic responses. Pediatr Crit Care Med.

